# Fibroblast activation protein (FAP) as a prognostic biomarker in multiple tumors and its therapeutic potential in head and neck squamous cell carcinoma

**DOI:** 10.32604/or.2024.046965

**Published:** 2024-07-17

**Authors:** RUIFANG LI, XINRONG NAN, MING LI, OMAR RAHHAL

**Affiliations:** 1 School of Stomatology, Shanxi Medical University, Taiyuan, 030001, China; 2 Department of Stomatology, The First Hospital of Shanxi Medical University, Taiyuan, 030001, China; 3 Department of Oral and Maxillofacial Surgery, Xiangya Stomatology Hospital, Central South University, Changsha, 410005, China

**Keywords:** FAP, Head and neck squamous cell carcinoma, Cancer, Prognosis, Tumor microenvironment, Molecular function

## Abstract

**Background:**

Fibroblast activation protein (FAP), a cell surface serine protease, plays roles in tumor invasion and immune regulation. However, there is currently no pan-cancer analysis of FAP. **Objective:** We aimed to assess the pan-cancer expression profile of FAP, its molecular function, and its potential role in head and neck squamous cell carcinoma (HNSC).

**Methods:**

We analyzed gene expression, survival status, immune infiltration, and molecular functional pathways of FAP in The Cancer Genome Atlas (TCGA) and Genotype Tissue Expression (GTEx) tumors. Furthermore, to elucidate the role of FAP in HNSC, we performed proliferation, migration, and invasion assays post-FAP overexpression or knock-down.

**Results:**

FAP expression was elevated in nine tumor types and was associated with poor survival in eight of them. In the context of immune infiltration, FAP expression negatively correlated with CD8+ T-cell infiltration in five tumor types and positively with regulatory T-cell infiltration in four tumor types. Our enrichment analysis highlighted FAP’s involvement in the PI3K-Akt signaling pathway. In HNSC cells, FAP overexpression activated the PI3K-Akt pathway, promoting tumor proliferation, migration, and invasion. Conversely, FAP knockdown showed inhibitory effects.

**Conclusion:**

Our study unveils the association of FAP with poor tumor prognosis across multiple cancers and highlights its potential as a therapeutic target in HNSC.

## Introduction

Cancer represents a major public health concern worldwide, with its intricate etiology stemming from various genetic and environmental factors. Given the complexity of tumorigenesis and tumor microenvironment, pan-cancer analysis of any potentially oncogenic gene is necessary. Tumorigenesis is influenced by many factors, including oncogene activation, immune escape, and abnormal signaling pathway activation [1,2]. It is worth noting that most well-known oncogenes are abnormally expressed and play an essential role in many types of tumors [3]. Therefore, pan-carcinoma analysis is more effective in scientifically assessing a crucial oncogene in tumor progression. The clinicopathological data in common databases (TCGA and GTEx) are available, and it is essential to use them to evaluate the potential oncogene.

FAP is a type II transmembrane serine protease, almost exclusively expressed in pathological conditions such as fibrosis, arthritis, and cancer, which was first identified in cultured fibroblasts by Wolfgang Rettig in 1986 and described as a cell surface antigen. In 1990, FAP was proved to be a cell surface serine protease and could be shed from the plasma membrane forming a soluble FAP [4,5]. The expression of FAP is upregulated in more than 90% of epithelial malignant tumor cells but not in normal tissues of adults [6]. Evidence suggests that FAP is involved in cancer development through its enzymatic or non-enzymatic activity [7]. The substrates of FAP protein mainly are type I and type III collagen [8]. The non-enzymatic function of FAP was found in a breast cancer study, transfection with catalytically inactive FAP in breast cancer cell lines enhanced cell proliferation, migration, and invasion abilities, suggesting that the non-enzymatic activity of FAP also was involved in tumor progression [9]. Recent studies highlight that the overexpression of FAP in the stomach, colorectal, and breast cancer was associated with tumor growth and metastasis [10–12]. Whether FAP plays the same role in other tumors is worth further investigating. Therefore, we performed a pan-cancer analysis and verified the potential carcinogenic effects of FAP in HNSC.

Our study first uses the common databases for a pan-carcinomatological analysis of FAP, including gene expression, survival status, immune infiltration, and FAP-related molecular functions. In addition, we focused on analyzing the clinical significance of FAP in HNSC patients and its effects on the proliferation, migration, and invasion of HNSC cells.

## Materials and Methods

### Gene expression analysis and survival prognosis analysis

The data on FAP gene expression was obtained from the Gene Expression Profiling Interactive Analysis (GEPIA) web to get the expression difference between the TCGA cancer types and their corresponding normal tissues [13]. Additionally, we accessed the FAP expression of different pathological stages in TCGA tumors via the GEPIA2 web.

Subsequently, using the The University of ALabama at Birmingham CANcer data analysis Portal (UALCAN) web to validate the differential expression of FAP protein between tumor and paired normal tissues [14].

The GEPIA2 was also used to analyze the correlation between Overall survival (OS) and expression of FAP from all TCGA tumors. Based on the TCGA database, the receiver operator characteristic curve (ROC) was used to calculate the predictive efficacy of FAP expression for the overall survival of HNSC patients.

### Immune infiltration analysis of FAP

The TIMER2 website explored the association between FAP expression and immune infiltrates in 33 cancer types [15]. The CD8+ T cells and T regulatory cells were selected. Estimating the Proportion of Immune and Cancer cells (EPIC, Based on gene expression data, the proportion of immune cells and tumor cells present in the tumor sample is inferred), Microenvironment Cell Populations counter (MCPcounter/MCPCOUNTER, Estimating the population abundance of tissue-infiltrating immune and stromal cell populations using gene expression), quantification of the Tumor Immune contexture from human RNA-seq data (quanTIseq/QUANTISEQ, Based on human RNA-seq data, the proportion of ten different immune cell types present in the sample and the proportion of other uncharacterized cells were quantified by deconvolution), CIBERSORT (A deconvolution algorithm was used to estimate the composition and abundance of immune cells in the mixed cells based on transcriptome data), and XCELL (The expression characteristics of immune cells were enriched and analyzed, and converted into scores of corresponding cell types) were used to analyze immune infiltration. Most algorithms determined the final correlation between FAP expression and immune infiltrates with a significant correlation (*p* < 0.05).

### FAP-related gene enrichment analysis

This study first used the STRING website to analyze the interactional proteins of FAP in the organism of “Homo sapiens” [16]. The network diagram showed the relationship between FAP and its interacted proteins.

Subsequently, the top 100 FAP co-expressed upregulated and down-regulated genes based on TCGA datasets of 33 cancer types were collected for enrichment analysis. This study combined FAP co-expressed and FAP-interacted genes to perform KEGG pathway analysis via the Database for Annotation, Visualization and Integrated Discovery (DAVID) website [17,18]. Bubble diagrams of KEGG pathways were generated using R software, with thresholds set at ‘gene count greater than or equal to five’.

### Cell lines, cell culture conditions, and transfection

The Human oral keratinocytes (HOK) and HNSC cell lines SCC9, SCC15, HN4, SCC25, and CAL27 were provided by the CCTCC. All these cells were cultured in Dulbecco’s Modified Eagle’s Medium (DMEM) (Biological Industries, Kibbutz Beit Haymaker, Israel) with 4500 mg/L glucose containing 10% heat-inactivated FBS (Biological Industries, Kibbutz Beit Haymaker, Israel), 100 μg/ml streptomycin (NCM Biotech, Suzhou, China) and 100 U/ml penicillin (NCM Biotech, Suzhou, China), cultured in a cell incubator (Reward Life Technology, Shenzhen, China) containing 5% carbon dioxide at 37°C. The overexpression and RNAi plasmid was constructed according to the reference sequence of FAP (NM_001291807.3) in the National Center for Biotechnology Information (NCBI) database. The overexpression plasmid was established on the vector of GV272. The RNAi plasmid was shown on the vector of GV248. Transient transfection was completed by Lipofectamine 3000 (Invitrogen, California, USA).

### RNA extraction and real-time PCR analysis

Cells or tissues were lysed using TRIzol reagent (Bioss, Beijing, China) and the lysate was transferred to centrifuge tubes (Corning, New York, USA). Then, trichloromethane and isopropanol (Bioss, Beijing, China) were used to separate and purify the extracts. Finally, the precipitated RNA was washed with absolute ethanol. cDNA was synthesized using the HiScript II QRT SuperMix for qPCR reagent Kit (Vazyme, Nanjing, China). All qPCR reactions were performed using ChamQ Universal SYBR qPCR Master Mix (Vazyme, Nanjing, China). The amplified cDNA was produced with reaction conditions of 95°C, 30 s; 95°C, 10 s; 60°C, 30 s; a total of 40 cycles—the quantified and normalized results using GAPDH as a control. The primer sequence of the FAP gene was 5′-GAAAGACGGGGGACTGACTT-3′ (Forward) and 5′-GCTGCAAGGACCATACA-3′ (Reverse). The source and use of the samples involved in this assay have been approved by the Ethics Committee of Xiangya Stomatology Hospital of Central South University (20200092). All samples collection and processing were carried out respecting the Declaration of Helsinki. All patients signed informed consent prior to tumor tissues collection treatment.

### Protein extraction and western blot analysis

When the cell density reached 85%–95%, 120 μL of cell lysis buffer (Bioss, Beijing, China) containing protease inhibitor was added. Protein samples (30 μg) were resolved on 6% or 8% polyacrylamide gels (Vazyme, Nanjing, China) and transferred to 0.45 μm Polyvinylidene Fluoride (PVDF) membranes (Bitai Biotechnology, Shanghai, China) by electroblotting. The membranes were blocked with 4% bovine serum albumin (Vazyme, Nanjing, China) for 12 h at 4°C temperature and incubated with primary antibodies. The primary antibodies used in this western blot assay were Anti-FAP (Abcam, Cambridge, UK, 1:1000), Anti-PI3K (Abcam, Cambridge, UK, 1:1000), Anti-p-PI3K (Abcam, Cambridge, UK, 1:1000), Anti-Akt1+Akt2+Akt3 (Abcam, Cambridge, UK, 1:1000), Anti-p-Akt1+Akt2+Akt3 (Abcam, Cambridge, UK, 1:1000), and GAPDH (Bioss, Beijing, China, 1:1000). After overnight culture, the primary antibody was washed three times with Ttris Buffered saline with Tween-20 (TBST, Vazyme, Nanjing, China, 1×) for 10 min each time, and then the secondary antibodies labeled with HRP (Bioss, Beijing, China; 1:10,000) were added and cultured for 1 h at room temperature.

### Cell proliferation analysis

After successful transfection of FAP overexpressing or knock-down plasmid (Jikai Gene, Shanghai, China), tumor cells were cultured in 96-well plates with 1000 cells per well and 100 μL complete medium per well. Cell Counting Kit-8 (Dojindo, Shanghai, China) was used to detect the optical density value of each well of the culture plate at each time point. Each detection was performed with 100 μL premixed solution (90 μL complete medium and 10 μL CCK-8 reagent). The growth velocity of tumor cells was seen by a Microplate Reader System (Tanon, Minnesota, USA) with 0, 12, 24, 48, 72, and 96 h.

### Wound-healing assay

The experimental procedure refers to our previous research [19]. Tumor cells were evenly spread on 6-well plates (Corning, New York, USA) and cultured until confluent. After starvation treatment with serum-free DMEM medium for six hour, cells were scraped with a 10 μL pipetting tip (time 0), washed with PBS, and incubated with serum-free DMEM. Five non-overlapping field photos were randomly taken at 24 h.

### Transwell migration and invasion assay

Transwell chambers (Corning, New York, USA), each with a pore size of 0.8 μm, were added to 24-well plates, and the upper surface of the bottom of the chamber was coated with Matrigel (Becton, Dickinson and Company, New York, USA) gum for cell invasion assay. After tumor cells were routinely digested and counted, they were prepared into single-cell suspension with serum-free DMEM and the concentration was adjusted to 1 × 105/mL. 300 μL single-cell suspension was placed above the Transwell chamber and 700 μL DMEM medium containing 20% serum was placed below Transwell chamber. After 24 h, cells passing through the bottom of the chamber were fixed with paraformaldehyde (Vazyme, Nanjing, China), stained with crystal violet (Vazyme, Nanjing, China), and counted.

### Statistical analysis

All statistical analyses were performed using SPSS 22.0 software unless otherwise stated. Kaplan-Meier analysis was used to evaluate survival outcomes of patients with differing FAP expression levels, with significance tested using the log-rank test. Paired sample *t*-test was employed to compare the means of two related groups. Univariate and multivariate Cox regression analyses were performed to investigate the association between FAP expression and various clinicopathological factors of HNSC patients in the TCGA database. The hazard ratio (HR) with a 95% confidence interval (CI) was measured to estimate the hazard risk of individual factors. A nomogram, a graphical representation of the prediction model, was constructed using the R language to evaluate the prognostic significance of FAP expression. A *p*-value less than 0.05 was considered statistically significant. All *p*-values reported were two-tailed.

## Results

### The gene expression and survival analysis of FAP

**Figure 1:**
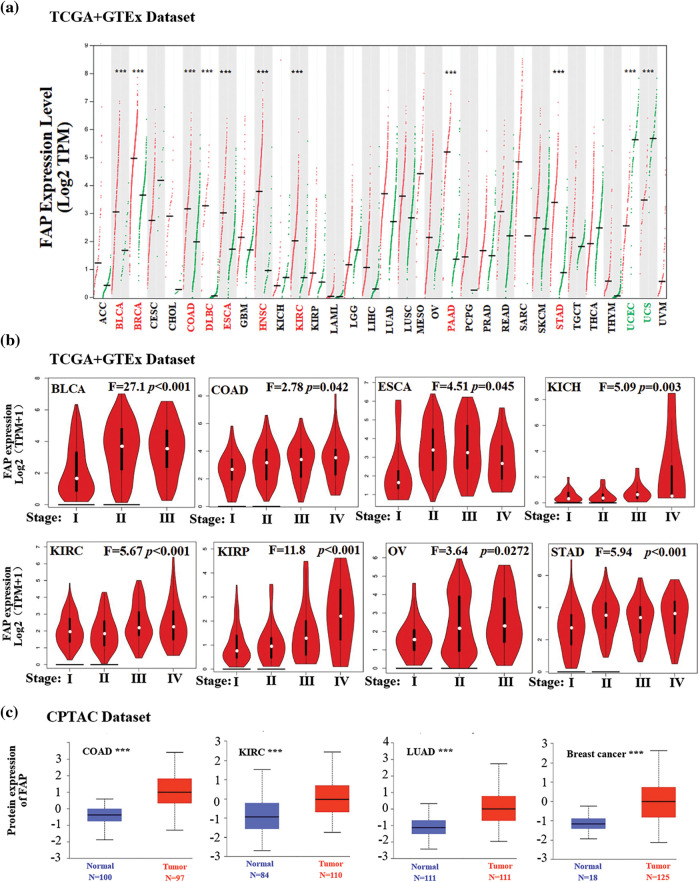
Expression level of FAP in different tumors. (a) The expression status of the FAP gene in different cancers was analyzed through GEPIA. (b) Expression level of FAP gene in different pathological stages of BLCA, COAD, ESCA, KICH, KIRC, KIRP, OV, and TGCT were analyzed. (c) Based on the CPTAC dataset, the expression level of FAP total protein between normal tissue and tumor tissue of COAD, KIRC, LUAD, and breast cancer were analyzed. ***p < 0.001.

We investigated the differential expression of FAP in various tumor types and its correlation with survival. FAP was upregulated in several cancer types such as Bladder urothelial carcinoma (BLCA), Breast invasive carcinoma (BRCA), and Colon adenocarcinoma (COAD), Lymphoid neoplasm diffuse large B-cell lymphoma (DLBC), Esophageal carcinoma (ESCA), HNSC, Kidney renal clear cell carcinoma (KIRC), Pancreatio adenocarcinoma (PAAD), and Stomach adenocarcinoma (STAD) compared to match normal tissues (Fig. 1a). However, in Uterine *corpus* endometrial carcinoma (UCEC) and Uterine carcinosarcoma (UCS), FAP expression was reduced (Fig. 1a). The pathological stages of specific tumor types, including BLCA, COAD, ESCA, Kidney chromophobe (KICH), KIRC, Kidney papillary cell carcinoma (KIRP), Ovarian serous cystadenocarcinoma (OV), and STAD, correlated significantly with FAP expression (Fig. 1b). We verified FAP protein expression in select cancers through the UALCAN platform, observing elevated levels in tumors like COAD, KIRC, Lung adenocarcinoma (LUAD), and Breast cancer (Fig. 1c).

**Figure 2:**
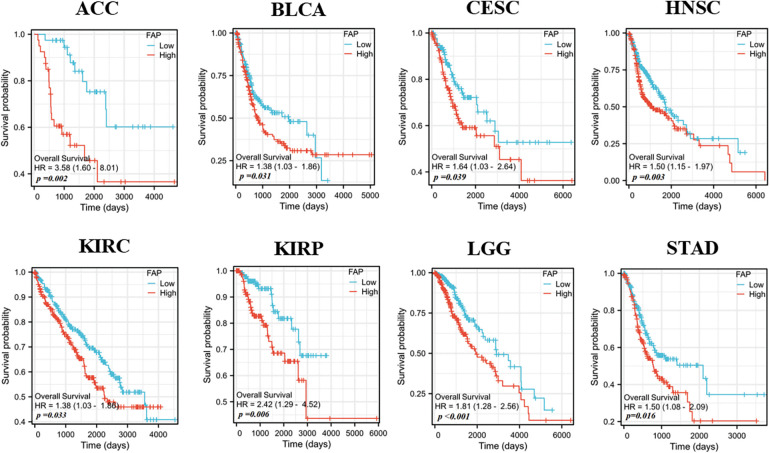
Relationships between FAP gene expression and overall survival prognosis of cancers in TCGA.

Increased FAP expression was associated with poor prognosis in cancers such as Adrenocortical carcinoma (ACC), BLCA, Cervical squamous cell carcinoma and endocervical adenocarcinoma (CESC), HNSC, KIRC, KIRP, Brain Lower Grade Glioma (LGG), and STAD (Fig. 2).

### Immune infiltration analysis of FAP

**Figure 3:**
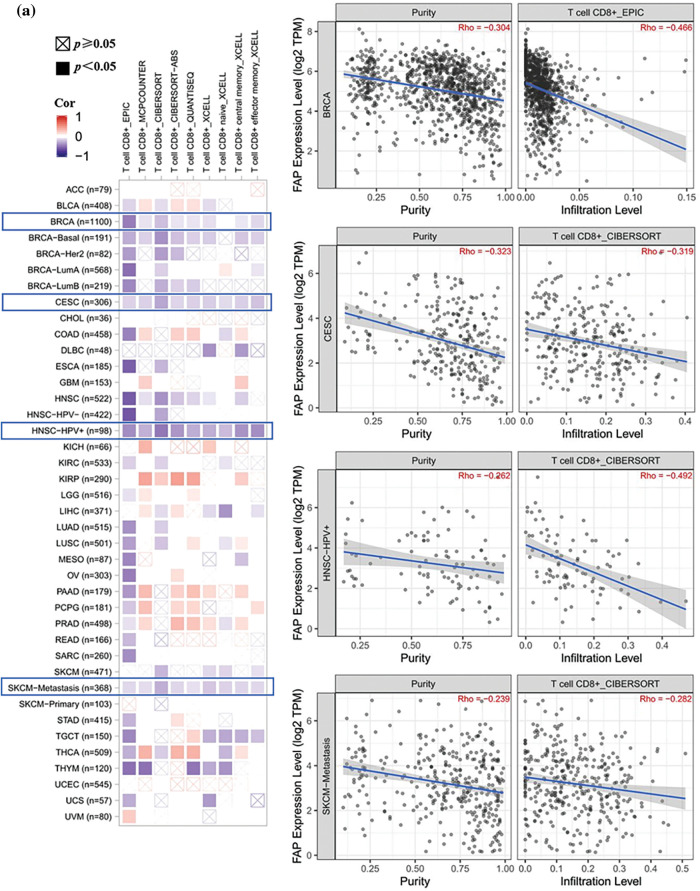

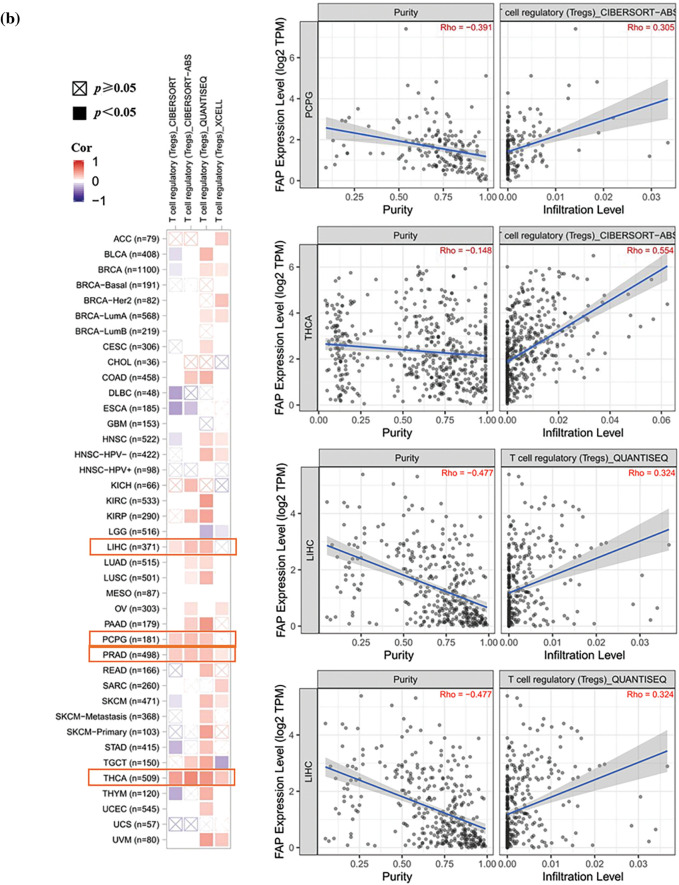
Different algorithms were used to explore the correlation analysis between FAP expression and immune infiltration. (a) Potential correlation between the expression level of FAP gene and the infiltration level of T CD8+ T cells from all types of cancer in TCGA. (b) The correlation between the expression level of the FAP gene and the infiltration level of regulatory T cells from TCGA cancers.

Given the pivotal role of immune infiltration in tumorigenesis [20], we examined the relationship between FAP expression and immune cell infiltration. High FAP expression negatively correlated with CD8+ T cell infiltration in tumors including BRCA, CESC, HNSC, and SKCM (Fig. 3a) but positively correlated with regulatory T cells in cancers such as LIHC, PCPG, PRAD, and THCA (Fig. 3b).

### FAP may involve the PI3K-Akt signaling pathway in HNSC

**Figure 4:**
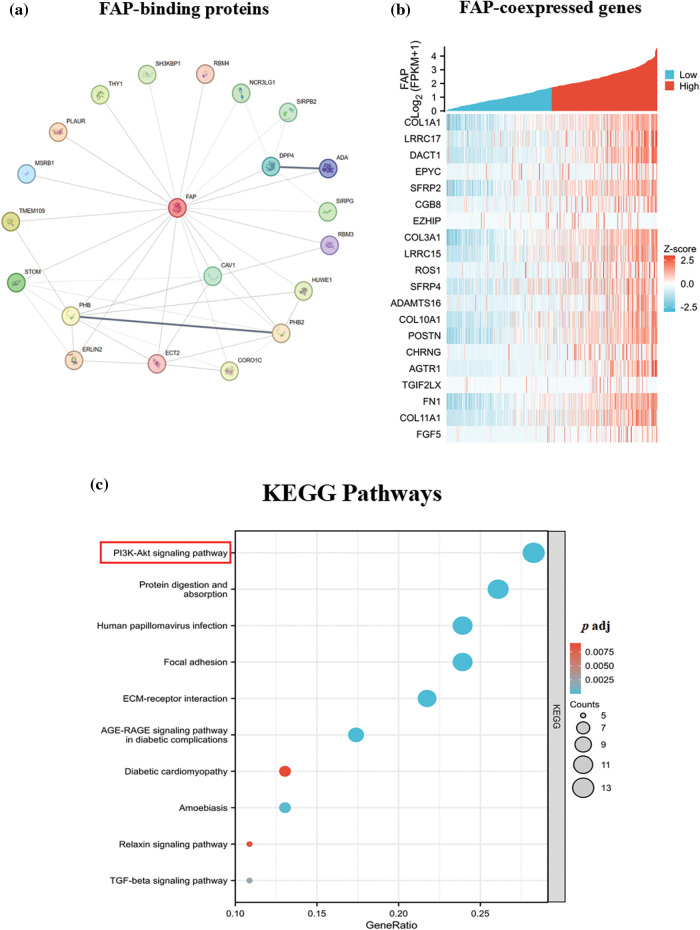
FAP-related gene enrichment analysis. (a) The experimentally determined top 20 FAP-binding proteins were abtained using the STRING tool. (b) The top 20 FAP co-expressed genes in TCGA were showed in heat map. (c) Based on the FAP-binding and co-expressed genes, KEGG pathway analysis was performed.

Utilizing the STRING tool, we identified 50 FAP-binding proteins (Fig. 4a). GEPIA2 web further revealed the top 20 FAP co-expressed genes (Fig. 4b). Enrichment analysis indicated FAP’s significant involvement in the PI3K-Akt signaling pathway (Fig. 4c).

### Relationship between FAP expression, clinicopathological factors and survival prognosis in HNSC patients

**Figure 5:**
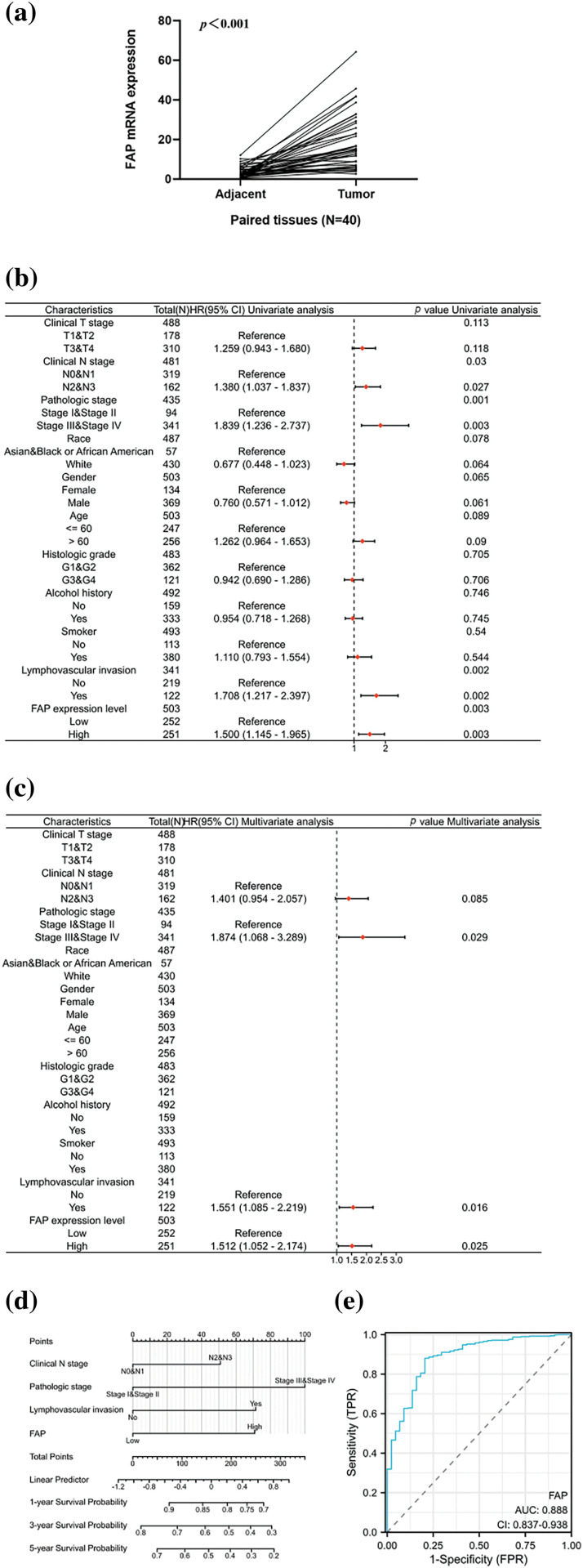
Overexpression of FAP was associated with poorer prognosis in HNSC patients. (a) The expression of FAP mRNA in 40 pairs of HNSC tissues and adjacent normal tissues. (b) Univariate analysis of FAP expression and also clinicopathological factors in TCGA patients with HNSC. (c) Multifactorial analysis of FAP expression and clinicopathological factors in TCGA patients with HNSC. (d) Nomogram for prognostic prediction. (e) The ROC curve of the nomogram predicting survival prognosis.

We evaluated FAP expression in 40 HNSC pairs, observing an upregulation in HNSC tissues (Fig. 5a). Subsequent analyses from TCGA revealed FAP expression, among other factors, as an independent prognostic indicator for HNSC patients (Figs. 5b and 5c). We developed a prognostic nomogram incorporating these factors to forecast patient survival (Fig. 5d), achieving an ROC value of 0.888 (Fig. 5e).

### FAP mediated the PI3K-Akt signaling pathway in HNSC

**Figure 6:**
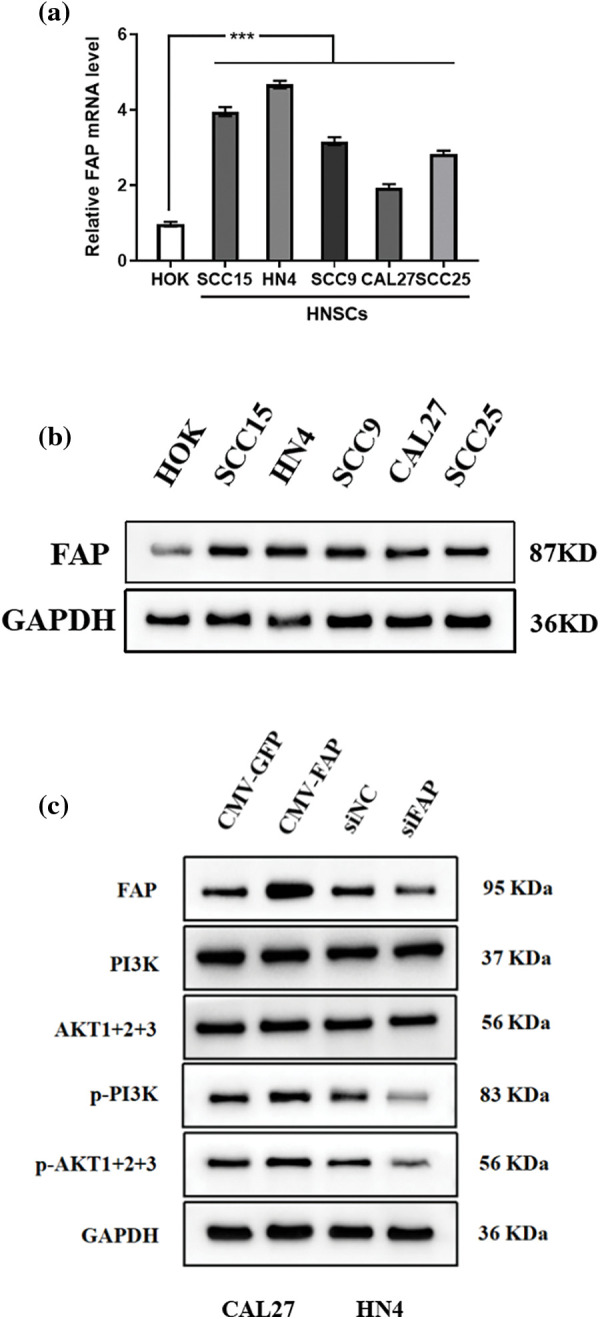
FAP mediated the PI3K-Akt signal pathway in HNSC cells. (a) The expression of FAP mRNA in HOK cell line and HNSCs cell lines (SCC15, CAL27, SCC9, HN4, and SCC25). (b) Western blotting was used to detect the expression of FAP protein in HOK and HNSC cells. (c) Western blot analysis of the expression of FAP and PI3K-Akt-related molecules with FAP overexpression or knock-down in HNSC cells. ***p < 0.001.

Comparing HNSC cell lines with normal oral epithelial cells, both FAP gene and protein levels were higher in HNSC cells (Figs. 6a and 6b). Furthermore, FAP overexpression upregulated PI3K-Akt pathway-related proteins in select cell lines, underscoring FAP’s role in this signaling pathway (Fig. 6c).

### FAP knock-down inhibited the proliferation, migration and invasion of HNSC cells

**Figure 7:**
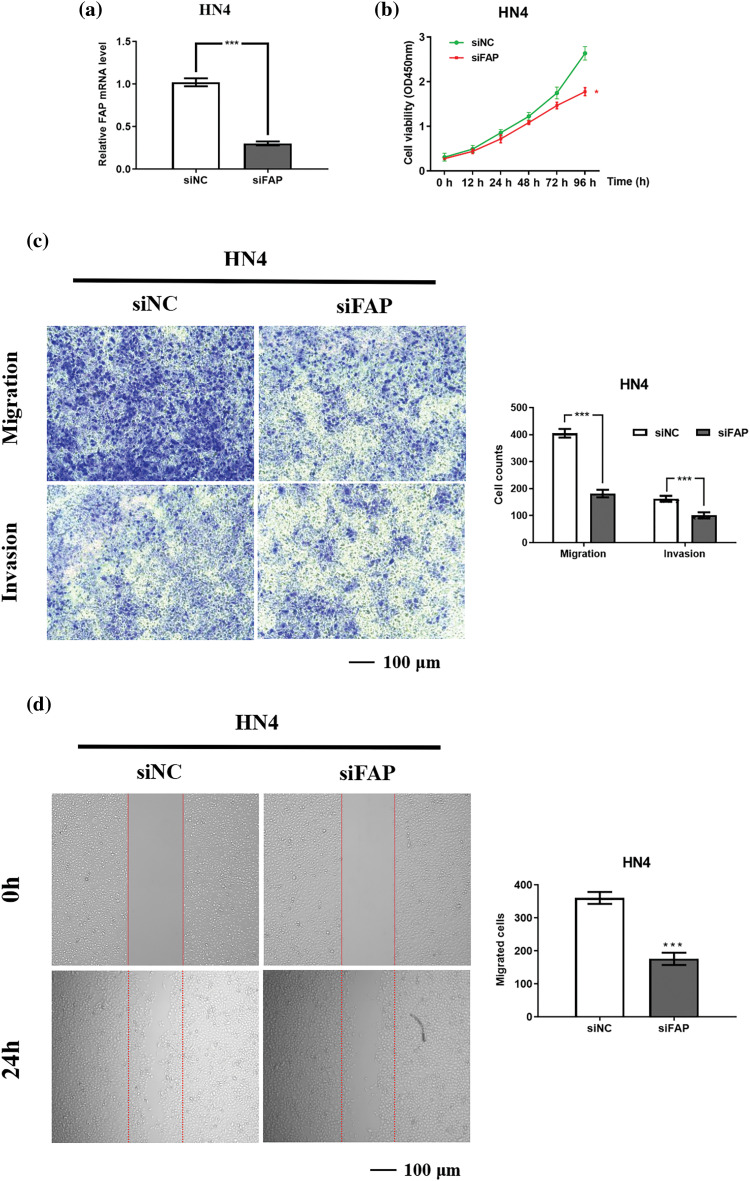
FAP overexpression promote the proliferation, migration and invasion of HNSC cells. (a) The results of real-time PCR showing FAP mRNA expression in HN4 cell after transfection of FAP knock-down vector for 48 h (***p < 0.001). (b) FAP knockdown in HN4 cells inhibited the the proliferative ability of tumor cells (**p < 0.01). (c) FAP knockdown in HN4 cells inhibited the migration and invasion of tumor cells (***p < 0.001). (d) FAP knockdown in HN4 cells inhibited the migration of tumor cells (***p < 0.001).

Reducing FAP expression in HN4 cells resulted in an approximate 75% decrease in its mRNA levels (Fig. 7a), accompanied by reduced cell proliferation, migration, and invasion (Figs. 7b–7d).

### FAP overexpression accelerated the proliferation, migration and invasion of HNSC cells

**Figure 8:**
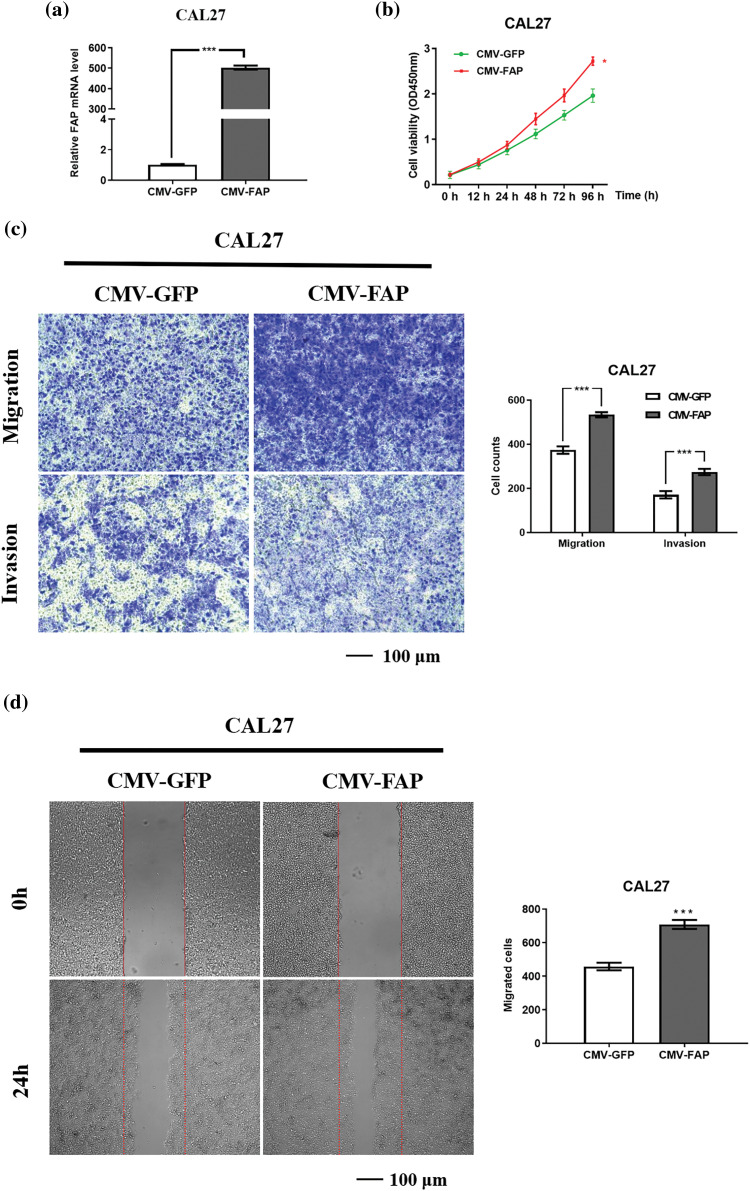
FAP knock-down inhibite the proliferation, migration and invasion of HNSC cells. (a) The expression of FAP mRNA in CAL27 cell after transfection of overexpression vector of FAP for 48 h (***p < 0.001). (b) Overexpression of FAP in CAL27 cells facilitated the proliferation were detected in tumor cells. (**p < 0.01). (c) Overexpression of FAP in CAL27 cells promoted the migration and invasion of tumor cells (***p < 0.001). (d) Overexpression of FAP in CAL27 cells facilitated the migration of tumor cells (***p < 0.001).

Elevating FAP expression in CAL27 cells led to a more than 200-fold mRNA increase (Fig. 8a). Such overexpression enhanced cell proliferation, migration, and invasion (Figs. 8b–8d).

## Discussion

It has been reported that the simple concept of FAP as an extracellular matrix degradation machine is no longer sufficient [21–23]. Oncogenes often have high expression and pro-cancer function in many types of tumors. Whether FAP could promote tumor progression through some specific molecular mechanisms remains to be further explored. According to the existing literature review, we could not find any literature on a pan-cancer analysis of FAP from a whole-tumor perspective. Thus, we analyzed the relationship between FAP expression and tumor staging, immune infiltration, and biological function of 33 tumors.

In this study, we found FAP was highly expressed in nine tumors in common databases, and overexpression of FAP was related to poor OS for ACC, BLCA, CESC, HNSC, KIRC, KIRP, LGG, and STAD. Recent studies suggest that FAP-targeted therapy can inhibit the proliferation of pancreatic tumor cells [24,25]. In gastric cancer, glioblastoma, and ovarian cancer, overexpression FAP is correlated with poor survival prognosis [26–28]. This study showed that FAP did not associate with the clinical prognosis of SKCM in TCGA database. Although the earliest survey of FAP came from melanoma, the expression and function of FAP in tumor progression remain unclear [29]. Furthermore, we analyzed the relationship between FAP expression and immune infiltration in all types of cancer. We found that FAP expression was negatively correlated with CD8+ T cell infiltration in BRCA, CESC, HNSC, and SKCM, positively correlated with regulatory T cells in LIHC, PCPG, PRAD, and THCA, which suggested that FAP may contribute to the inhibitory immune microenvironment in these cancers. The effects of FAP on immune infiltration began to be analyzed in recent years. Feig et al. [30] found that FAP could reduce tumor growth via CD4+/CD8+ T cell activity in PDAC. Yang et al. [31] also found that FAP+ cancer-associated fibroblasts (CAF) inhibited the differentiation of IFNγ+ CD8+ T cells compared with FAP-CAF cells. Our findings first presented the positive correlation between FAP expression and the immune infiltration level of regulatory T cells in LIHC, PCPG, PRAD, and THCA.

Finally, to clarify the molecular role of FAP in cancer progression, this study first integrated the information on FAP-binding and FAP co-expressed genes across all tumors for a series of enrichment analyses and identified the potential carcinogenic mechanism of FAP. We found that FAP may promote tumor progression via the “PI3K-Akt” signal pathway. We further verified these bioinformatics results in HNSC. We found that FAP was one of the independent predictors of survival prognosis in HNSC patients and could promote proliferation, migration, and invasion of HNSC cells via the PI3K-Akt pathway. Wen et al. [22] also found that pancreatic stellate cells with FAP overexpression could promote the migration and invasion of pancreatic cancer via Akt phosphorylation. In addition, Jia et al. [32] found that FAP could promote the proliferation and migration of lung cancer cells via the PI3K-Akt signal pathway. In oral submucosal fibrosis (OSF), FAP promotes the proliferation, migration, and activation of oral fibroblasts via the PI3K-Akt signaling pathway [33]. There are some limitations in our study. The carcinogenic mechanism of FAP has not been validated in multiple TCGA tumors and has not been further confirmed *in vivo*.

Taken together, our first designed a pan-cancer analysis of FAP with clinical prognosis, immune cell infiltration, genetic mutation, and molecular function in 33 types of cancer. Then, we demonstrated that FAP might contribute to tumor progression of HNSC through the PI3K-Akt signaling pathway, which provided the reference for understanding the role of FAP in tumor progression and its value in targeted therapy.

## Data Availability

The datasets used and/or analyzed during the current study are available from the corresponding author on reasonable request.
